# Effects of Optogenetic Stimulation of Primary Somatosensory Cortex and Its Projections to Striatum on Vibrotactile Perception in Freely Moving Rats

**DOI:** 10.1523/ENEURO.0453-20.2021

**Published:** 2021-03-05

**Authors:** Zongpeng Sun, Artur Schneider, Mansour Alyahyay, Golan Karvat, Ilka Diester

**Affiliations:** 1 Optophysiology Lab, Institute of Biology III, University of Freiburg, Freiburg 79104, Germany; 2 BrainLinks-BrainTools, Intelligent Machine-Brain Interfacing Technology, University of Freiburg, Freiburg 79104, Germany; 3 Bernstein Center Freiburg, University of Freiburg, Freiburg 79104, Germany

**Keywords:** corticostriatal pathway, forelimb, optogenetics, primary somatosensory cortex, striatum

## Abstract

Tactile sensation is one of our primary means to collect information about the nearby environment and thus crucial for daily activities and survival. Therefore, it is of high importance to restore sensory feedback after sensory loss. Optogenetic manipulation allows local or pathway-specific write-in of information. However, it remains elusive whether optogenetic stimulation can be interpreted as tactile sensation to guide operant behavior and how it is integrated with tactile stimuli. To address these questions, we employed a vibrotactile detection task combined with optogenetic neuromodulation in freely moving rats. By bidirectionally manipulating the activity of neurons in primary somatosensory cortex (S1), we demonstrated that optical activation as well as inhibition of S1 reduced the detection rate for vibrotactile stimuli. Interestingly, activation of corticostriatal terminals improved the detection of tactile stimuli, while inhibition of corticostriatal terminals did not affect the performance. To manipulate the corticostriatal pathway more specifically, we employed a dual viral system. Activation of corticostriatal cell bodies disturbed the tactile perception while activation of corticostriatal terminals slightly facilitated the detection of vibrotactile stimuli. In the absence of tactile stimuli, both corticostriatal cell bodies as well as terminals caused a reaction. Taken together, our data confirmed the possibility to restore sensation using optogenetics and demonstrated that S1 and its descending projections to striatum play differential roles in the neural processing underlying vibrotactile detection.

## Significance Statement

The capability of writing-in information locally or pathway-specific makes optogenetic manipulation a promising approach to restore sensation. The extent to which the optogenetic stimulation can be interpreted as tactile sensation to guide operant behavior remains to be clarified. In this work we applied bidirectional manipulation of primary somatosensory cortex (S1) and its striatal terminals as well as pathway-specific activations, and found that cortical manipulations disturbed the performance of vibrotactile stimuli. Corticostriatal terminal activation enhanced the performance in the presence of vibrotactile stimuli, while corticostriatal inhibition did not affect the performance. This study provides insights into the contributions of S1 and its descending projections to striatum to the neural processing underlying vibrotactile detection.

## Introduction

Tactile sensation is one of the main information sources about the nearby environment. Vibration perception, as a part of tactile perception and an ancient mode of sensation, is used for many purposes, including identifying the texture of surfaces, mating, and feeling the approaches of predators which in turn helps to escape from them (Hill, 2001; Mazzoni et al., 2013; Polajnar et al., 2014; Noguera and Velando, 2019; Virant-Doberlet et al., 2019). However, it remains poorly understood whether it is possible to deliver artificial sensory information via optogenetics and how this might interfere with the perception of real tactile stimuli.

Several studies addressed the question of how to generate artificial sensation in sensory areas (Romo et al., 1998; Bensmaia and Miller, 2014) to convey sensory feedback and thereby to improve the performance of brain-machine interfaces (BMIs). For instance, the development of brain-machine-brain interfaces (BMBIs) using intracortical microstimulation (ICMS) of sensory cortex in monkeys have been demonstrated to be effective in enhancing prosthetic sensation (O'Doherty et al., 2011; Medina et al., 2012). Optogenetics as an alternative to electrical microstimulation has several advantages over traditional electrical stimulation, e.g., the option for bidirectional stimulation (i.e., activation or inactivation of neuronal activity).

Optogenetic stimulation has already been reported to be able to evoke different sensations. For example, optogenetic stimulation was applied to the barrel cortex of a mouse thereby inducing an illusory touch (Huber et al., 2008; O'Connor et al., 2013). A recent study revealed that the artificial sensation induced by optogenetic stimulation can be used to guide learning, which highlights the functional potential of optogenetic stimulation (Prsa et al., 2017). In non-human primates optical stimulation in primary somatosensory cortex (S1) can trigger responses (May et al., 2014). However, it remains unknown which neuronal elements are best suited for manipulating perception. Here, we address this question by selectively manipulating cortical neurons, corticostriatal projection neurons, and corticostriatal terminals. We decided to look into the corticostriatal connection since S1 sends direct and strong projections to striatum (Wall et al., 2013; Reig and Silberberg, 2014; Sippy et al., 2015; Hintiryan et al., 2016; Hidalgo-Balbuena et al., 2019).

## Materials and Methods

### Animals

Female Sprague Dawley (*n *=* *11) rats were included in this study. Before implantation, they were group-housed on a reverse 12/12 h light/dark cycle. To protect the implants, they were single-housed after surgery. During the course of the experiment, animals were maintained at no less than 80% of their free-feeding body weight. Rats were water deprived before the experiment and they had *ad libitum* access to food throughout the duration of the entire experiment. Free water was provided 2 d per week. Animal procedures were conducted in accordance with the guideline RL 201063 EU, and were approved by the University of Freiburg.

### Apparatus and behavioral procedures

Behavioral experiments were conducted in a 30 × 25 × 30 cm Plexiglas chamber equipped with a lever and a reward port. The lever was mounted in the middle of the front wall 1 cm above the floor. A 3D-printed rod (7 mm in diameter) was mounted on the lever to allow the animal to place its forepaw on it. A reward delivery port was on the right wall and connected to a syringe pump (Med Associates) located outside the sound-attenuating box. Two 6-cm-high walls were mounted on the left and right side of the lever. The walls extended further into the chamber than the lever and were 2 cm apart from each other to ensure that the rats manipulated the lever with their paws, while preventing them from using their mouths. A cue light that indicated the initiation of the trial and a house light were mounted on the right side outside the chamber. To introduce vibrations, a coin vibration motor (DC 3V, 10 × 2.7 mm, Seeed) glued to the lever was placed outside the chamber. A vibrator-attached control lever was mounted on the floor of the sound-attenuating box, to ensure that animals did not use the sound of the vibrator as an additional cue. Two LEDs (Conrad Electronic SE) of a wavelength similar to that of the laser (e.g., 473 and 594 nm) were placed in front of the chamber at the height of eyes of animals to mask the stimulation light.

**Figure 1.**
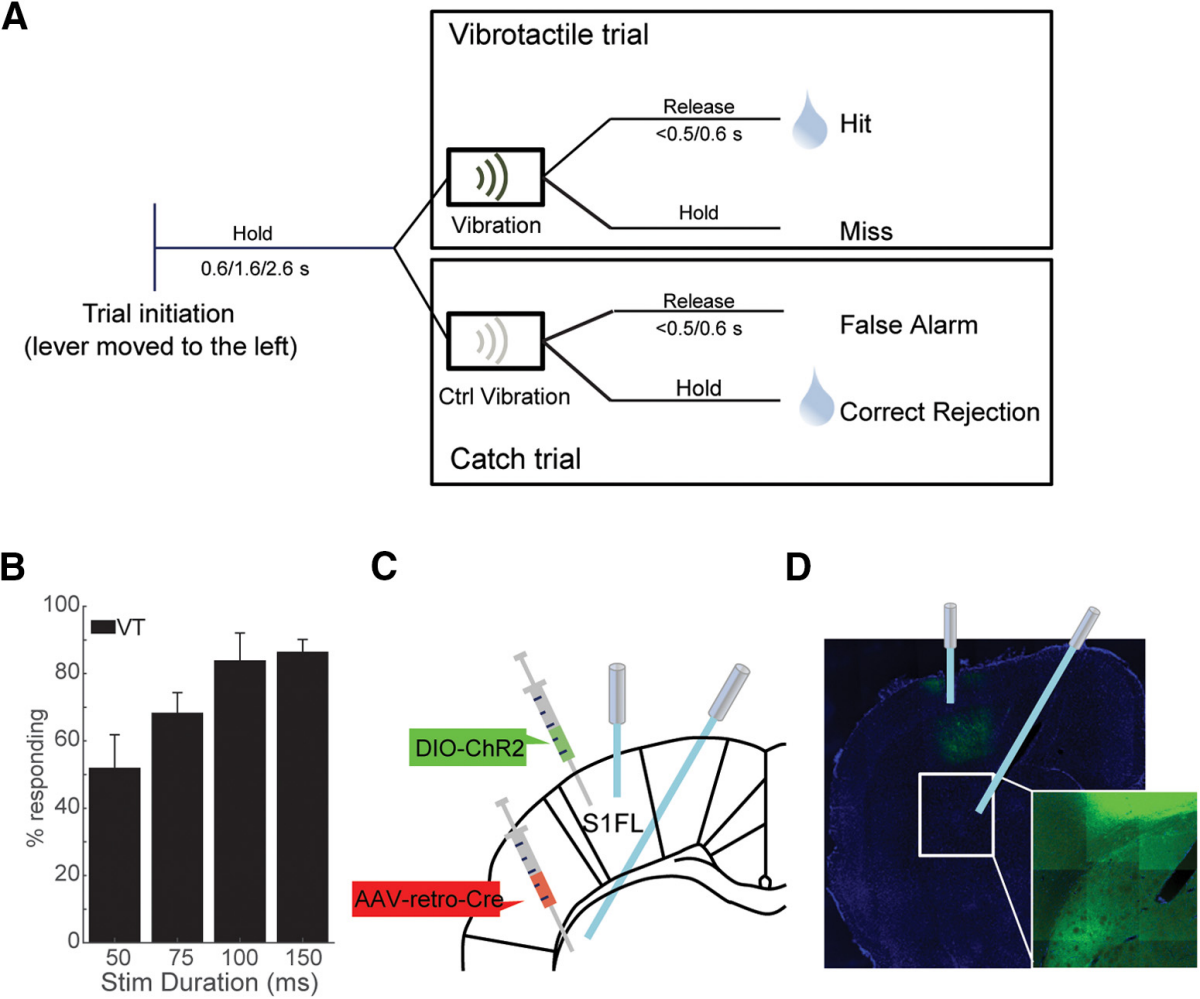
Schematic illustration of the experimental protocol. ***A***, In VTs, the rats had to release the lever within the allowed response window of 500 or 600 ms to receive a liquid reward. In CTs, the rats had to continue holding the lever when no vibration was delivered and were not rewarded if they released the lever within the response window. ***B***, Psychometric curve from an exemplary behavioral session. Data are given as mean ± SEM. ***C***, Schematic locations of optical fibers in striatum and S1. ***D***, Exemplary coronal sections of one rat injected with AAV5-EF1a-hChR2(H134R)-eYFP (S1FL) and AAV2-retro-Cre (striatum) viruses, respectively. Additional histologic evaluations are presented in Extended Data Figure 1-1. Scale bar: 1 mm. The inset represents an enlarged, higher magnification of the boxed region. Fibers are visible in striatum.

Rats were trained in the vibration detection task which required them to release the lever when they detected the vibration. The house light was switched on when a session started. When the lever was pulled to the left side by >7°, the cue light turned on to signal the initiation of the trial. 0.6, 1.6, or 2.6 s after the trial onset, a vibrotactile stimulus (with duration of 50, 75 100 or 150 ms) was delivered to the lever which instructed the animals to release the lever within 0.5 or 0.6 s after the onset of stimulus to get 3% sucrose solution (0.05 ml/s for 2 s) (Fig. 1). These three discreet holding durations were used to make the delivery of the vibrotactile stimulus unpredictable. To ensure that the animals perform the task based on the vibration rather than on other external cues, catch trials (CTs) in which the “control vibrator” was turned on instead of the “real vibrator” were pseudorandomly interleaved with vibrotactile trials (VTs). The control vibrator was attached to the control lever that was mounted on the floor outside the operant chamber, while the real vibrator was attached to the lever operated by the animals. In CTs, the animals had to hold the lever for a defined period after the stimulus. In the pathway-unspecific experiments, this response window was set to 0.5 s. In the pathway-specific manipulation experiment, to account for the putative onset delay of the vibrator, we increased the required additional holding period in CTs to 0.6 s. Error trials were followed by a 2-s timeout and white noise. Each session consisted of 50% real trials and 50% CTs. The animals had to achieve a success rate of at least 60%, which took four to six weeks, before being engaged in the optogenetic experiment.

### Surgery

The animals were anesthetized with a cocktail containing 80 mg/kg ketamine (Medistar) + 100 μg/kg medetomidine (Orion Pharma) intraperitoneally. Additional analgesics [10 mg/kg Carprofen (rimadyl, Zoetis) and 25 μg/kg Buprenorphine (Selectavet, Otto Fischer GmbH)] were administered subcutaneously. The skin around the surgical site of the head was shaved and cleaned thoroughly with Braunol (B. Braun Melsungen AG) followed by Kodan (Schülke). Rats were placed in a stereotaxic frame (David Kopf Instruments) with their eyes covered with ophthalmic ointment (Bepanthen, Bayer Health Care). The skin was incised, and tissue was removed with a bone scraper. Afterwards, a thin layer of super bond C&B cement (Sun Medical Co, LTD) was applied onto the skull, except the region for the viral injection and implantation. The position of head was adjusted until the height difference between bregma and λ was within 0.05 mm.

During the whole course of the surgery, the rats were placed on a heat pad to maintain the body temperature at 37°C, and their body temperature was monitored by using a rectal temperature sensor (Stoelting). Anesthesia was maintained with 2% isoflurane in oxygen (1 l/min) delivered through a face mask; 3-ml saline (0.9%, B. Braun Melsungen AG) was injected subcutaneously every 2 h to keep the rats hydrated.

For anterograde virus injection, a small craniotomy was made either bilaterally or unilaterally over the forelimb region of S1 (S1FL; Extended Data Fig. 1-1), then 0.5–1 μl AAV5-hSyn-hChR2(H134R)-eYFP or AAV5-hSyn-eNpHR3.0-mCherry viral vector (UNC Vector Core) was injected with a 10-μl gas-tight Hamilton syringe (World Precision Instruments) at a rate of 100 nl/min into the forelimb representation of the S1 (ML 4 mm, AP 0 mm from bregma) at a depth of 2 mm. In the case of pathway-specific stimulation, the AAV5-EF1a-hChR2(H134R)-eYFP viral vector (UNC Vector Core) was injected into S1FL in the same way. For the injection of AAV2-retro-Cre virus (UNC Vector Core) was injected into the striatum at the following coordinates: anteroposterior 0∼0.5 mm, mediolateral 3.5∼4 mm, dorsoventral −4.5 mm. For the viral injection into the striatum, we used a 30° angle to prevent from going through S1FL. To avoid back flow, the injection needle was left in the tissue for additional 10 min before being slowly extracted out of the brain. Immediately after viral vector injection, an optical fiber (200 μm in diameter, 0.37 NA, Doric lenses) with an external metal ferrule was implanted into S1FL and striatum. Fibers targeting striatum were implanted at a 20–30° angle such that the insertion targeting striatum does not go through S1.

10.1523/ENEURO.0453-20.2021.f1-1 Extended Data Figure 1-1 Histological verification of opsin expression in S1FL of a coronal slice from rats involved in optical activation (***A***), inactivation (***B***), and pathway-specific activation (**C**) experiments, respectively. Scale bar: 1 mm. The inset represents an enlarged, higher magnification of the boxed region. Fibers are visible in striatum. Download Figure 1-1, TIF file.

After applying Kwik-Cast Sealant (World Precision Instruments) over the craniotomy, we fixed the fibers to the skull using UV-cured dental cement (RelyX Unicem, 3 M ESPE) and secured them with several layers of Paladur dental cement (Heraeus). Water restriction was started at least 7 d after surgery when rats recovered from surgery. Analgesia (carprofen, 10 mg/kg, and buprenorphine, 25 μg/kg, i.p.) was administrated after the surgery for 3 d.

### Optogenetics and neural recording

Following one week of recovery from the surgery, the rats were water-restricted and retrained on the detection task to reach presurgery performance. Since the training took four to five weeks to reach a success rate of 60% and animals needed to adapt to the new physical condition after the surgery, presurgery and postsurgery training was required. The optogenetic stimulation commenced four weeks after viral injection. On each test day, the intensity of the laser from the 200-μm fiber patch cable (0.37 NA, Doric lenses) was measured with a PM100D optical power meter (Thorlabs Inc) to ensure that a stable light power (∼10 mW for photoactivation with 473 nm, and ∼15 mW for photoinactivation with 594 nm) was delivered.

Then the indwelling ferrule was tethered to the 200 μm fiber patch cable (0.37 NA, Doric lenses) which were coupled to the LightHUB compact laser combiner (OMICRON Laserage) via a fiber optic rotary joint included in the motorized ACO32 commutator (Tucker-Davis Technologies; TDT). For the ChR2 and corresponding controls for NpHR animals, blue laser light (λ = 473 nm) was delivered. For the NpHR and corresponding controls for ChR2 animals, yellow laser light (λ = 594 nm) was delivered. The continuous light whose duration was 100 ms longer than that of the corresponding vibration duration was delivered at the vibration onset. For the ChR2, the laser was on for 20% of trials. The laser was on for 50% trials in the case of NpHR.

**Figure 2.**
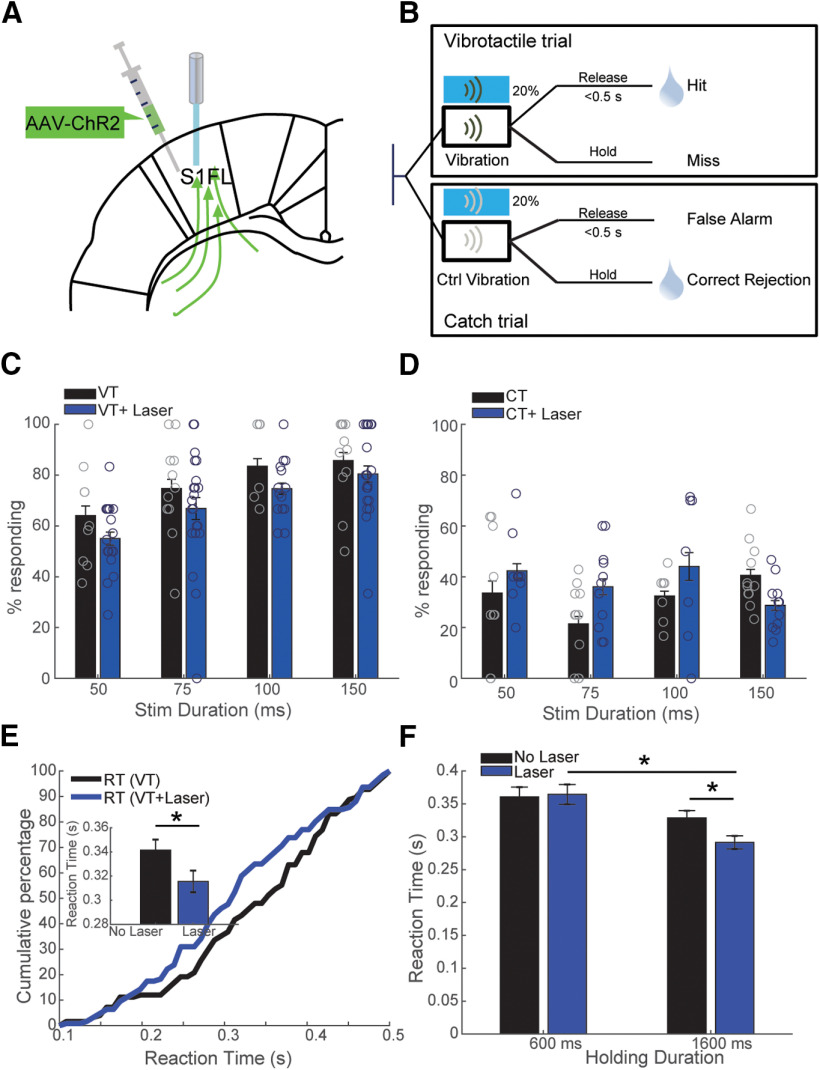
ChR2-mediated cortical optogenetic activation significantly compromised performance of rats and optogenetic activation reduced the RTs. ***A***, Schematic locations of viral injections and fiber implantations. ***B***, Time lines of events during VT and CT in the optoactivation experiment. Optogenetic manipulation was delivered after the rats hold the lever for 0.6 or 1.6 s. Laser was applied in 20% of a pseudorandomly selected subset of VTs and CTs. The duration of the laser on time was the same as that of vibrotactile stimuli. ***C***, ***D***, Behavioral performance for different stimulation durations. ***C***, The response rate as a function of stimulation duration for VT trials with (blue bars) and without laser stimulations (black bars). ***D***, The response rate as a function of stimulation duration for CT trials with (blue bars) and without laser stimulations (black bars). The effect of the duration of the optical S1 activation on RT is presented in Extended Data Figure 2-1*A*. The performance of the rats on pulsed blue laser stimulation is presented in Extended Data Figure 2-1*F*. ***E***, Cumulative probability of the RT of trials with laser (VT+laser, blue) of all durations and without laser (VT, black). Inset depicts the increase of RT caused by laser stimulation. A cumulative probability plot of the RT for different durations is presented in Extended Data Figure 2-1*B*,*C*. ***F***, Effect of laser stimulation on RT for trials with different holding durations. Data are mean ± SEM. The neural response analyses and the results of the control experiments are presented Extended Data Figure 2-1*D*,*E*.

**Figure 3.**
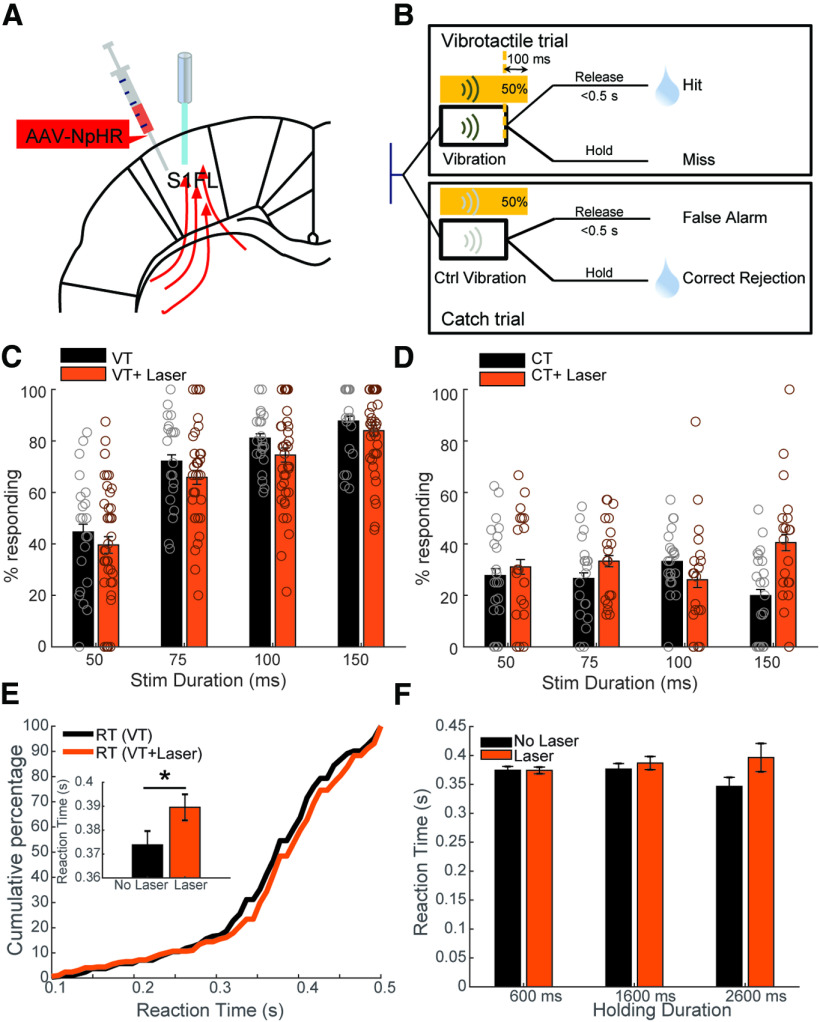
NpHR-mediated cortical optical inhibition impairs performance of rats and affects RTs (conventions are the same as in Fig. 2). ***A***, Schematic locations of viral injections and fiber implantations. ***B***, Time lines of events during VT and CT in the optoinhibition experiment. Optogenetic manipulation was delivered after the rats held the lever for 0.6, 1.6, or 2.6 s. Each session consisted of 50% VTs and 50% CTs (with control vibration). The yellow laser was turned on in 50% of pseudorandomly selected VTs and CTs. The duration of the laser on time was 100 ms longer than that of the tactile stimulus to compensate for the sensory delay. ***C***, ***D***, Assessment of performance changes induced by optical inhibition. ***C***, The response rate as a function of stimulation duration for VT trials with (yellow bars) and without laser stimulations (black bars). ***D***, The response rate as a function of stimulation duration for CT trials with (yellow bars) and without laser stimulations (black bars). The analysis of the time of lever release relative to control vibration, to laser off, and to “laser on” for different stimulation durations is presented in Extended Data Figure 3-1*D*. ***E***, Cumulative probability of the RT of trials with (yellow) and without (black) laser. Inset shows the increase of RT caused by laser stimulation of all durations. The effect of durations of optical S1 inhibition on RT is presented in Extended Data Figure 3-1*A–C*. ***F***, Effect of laser stimulation on RT for trials with different holding durations. Data are given as mean ± SEM.

**Figure 4.**
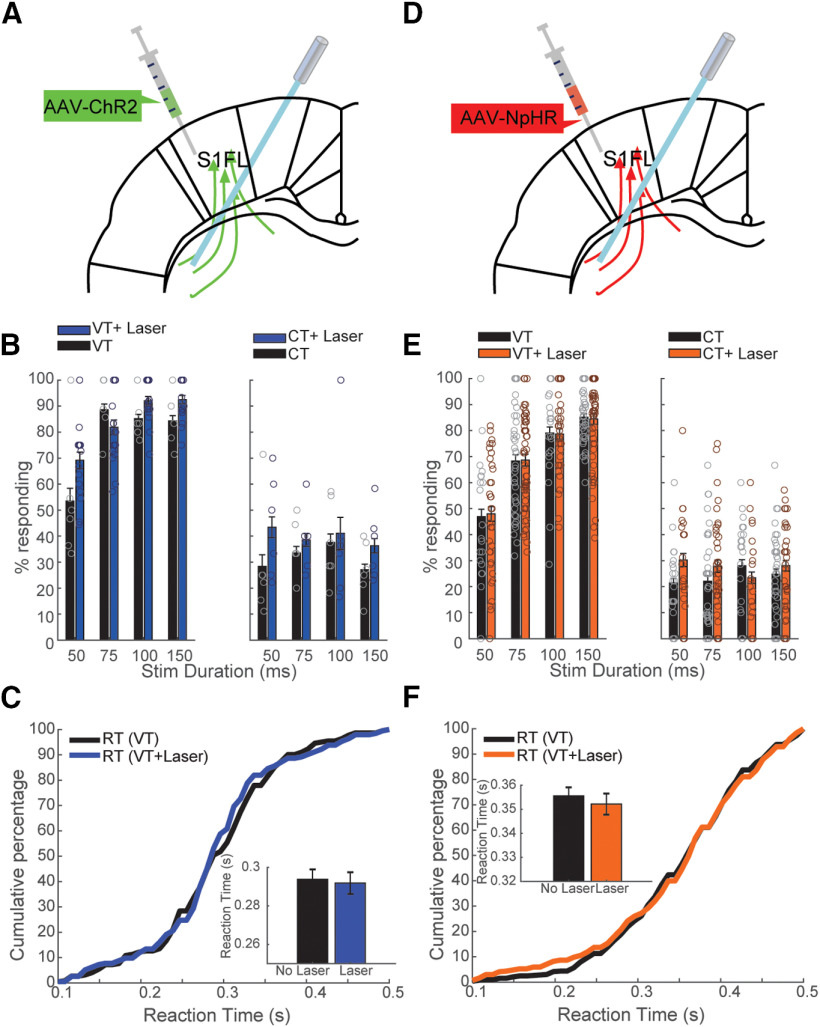
Effects of corticostriatal terminal optical activation (***A–C***) and inhibition (***D–F***) on performance. Data are given as mean ± SEM. ***A***, Schematic locations of viral injection location in S1FL and fiber implantation in striatum. ***B***, Changes in performance for the different stimulation durations. Effect of laser stimulation on the response rate as a function of stimulation duration for VT trials (left panel) and CT trials (right panel). ***C***, Cumulative probability of the RT of trials with tactile stimuli only (black) and trials with tactile stimuli and laser (blue). Inset depicts the decline of RT caused by laser stimulation. ***D–F***, Conventions are the same as ***A–C***.

**Figure 5.**
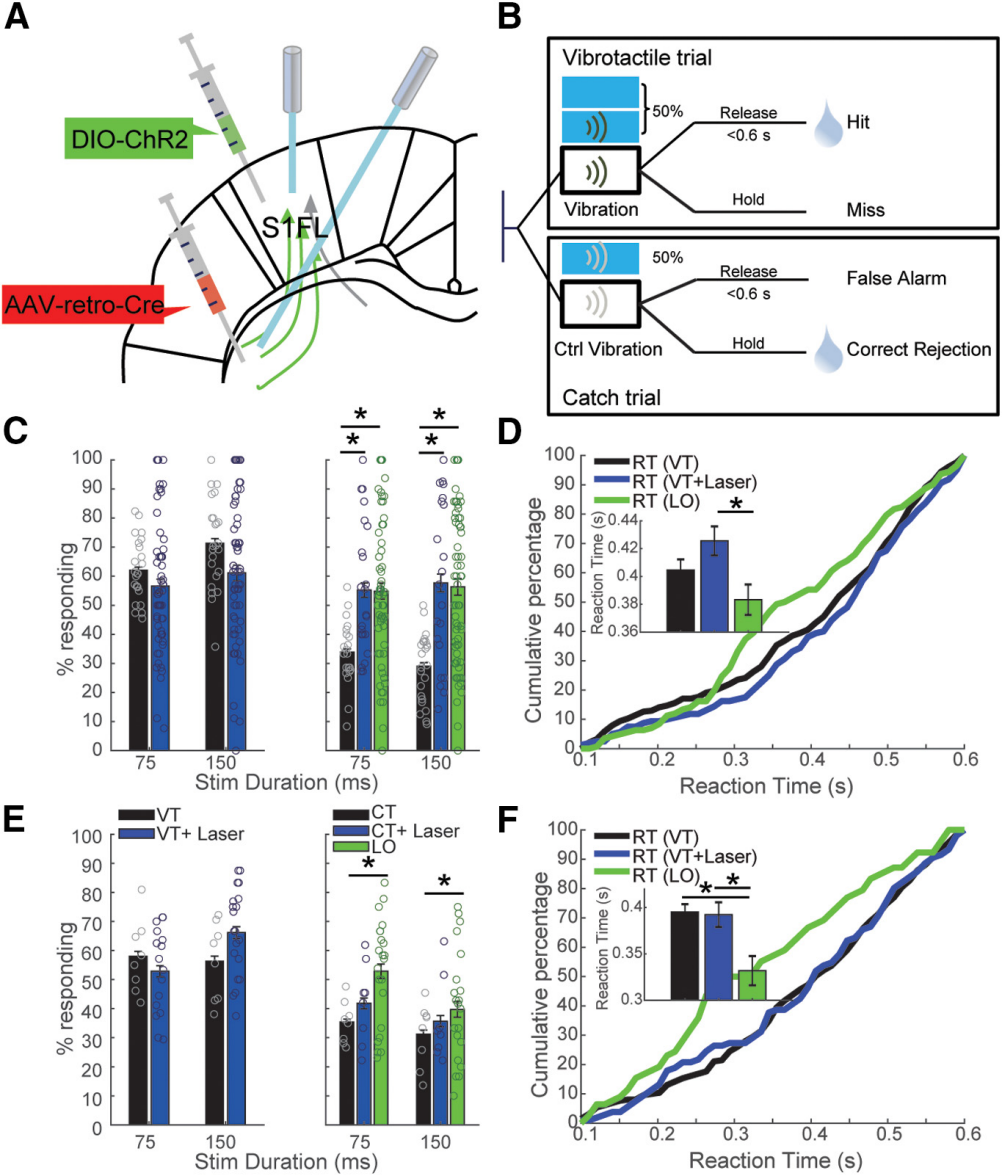
Optogenetic activation of corticostriatal neurons and corticostriatal terminals in striatum had differential effects on behavior. Data are given as mean ± SEM. ***A***, Schematic locations of viral injections in S1FL and striatum and fiber implantation in in S1FL and striatum. ***B***, Time lines of events during VT and CT in the pathway-specific optogenetic stimulation experiment; 50% of all trials were stimulated with laser. Within the 50% of stimulated trials in which responses to laser were rewarded, half of them contained only laser stimulation (LO), while the other half of stimulated trails contained both vibration and laser stimulation (VT+laser). ***C***, Performance changes induced by optogenetic activation of corticostriatal cell bodies in S1FL. Upper lines over bars indicate comparison between VT and LO, lower lines indicate comparison between VT and VT+laser for durations of 75 and 150 ms, respectively. ***D***, Cumulative frequency of the RT of trials without laser (VT, black), trials with laser and vibrotactile stimuli (VT+laser, blue), and trials with LO (green). ***E***, ***F***, Similar analysis for terminal activation in striatum. Extended Data Figure 5-1 shows that laser activation of S1 or corticostriatal neurons did not affect paw movement or locomotion.

We implanted optical fibers in S1FL of rats injected with AAV5-hSyn-hChR2(H134R)-eYFP (Fig. 2*A*). In optogenetic activation trials, a combination of real tactile stimulus and laser stimulation were delivered (VT+laser; Fig. 2*B*). Since responses to the optogenetic stimulation were not rewarded in CT+laser trials, laser stimulation was only applied in 20% of CTs to reduce confusion and frustration of the animals (Fig. 2*B*). For all optical activation experiments, the duration of the laser was the same as that of the tactile stimulus. In the optical inhibition experiment (Figs. 3 and 4), each session consisted of an identical amount of VTs and CTs. Optical inhibition was applied on 50% of VTs and CTs. For optogenetic silencing experiments, the laser was on continuously for 100 ms longer than the vibration to account for the time-delay for the sensory processing delay (Fig. 3). In the pathway-specific manipulation experiment (Fig. 5), only two stimulation durations (75 and 150 ms) were used, and 50% of all trials were stimulated with laser light. In half of stimulated trials, responses to laser were rewarded. Within 50% of these laser-rewarded stimulated trials, half of them contained only laser stimulation, while the other half of stimulated trails contained both vibration and laser stimulation.

10.1523/ENEURO.0453-20.2021.f2-1 Extended Data Figure 2-1 ***A–C***, Effect of durations of optical activation in S1on RT. ***A***, Effect of laser durations on RT. ***B***, ***C***, Cumulative probability plot of the RT of trials with tactile stimuli of different durations (***B***) and trials with both tactile stimuli and laser stimulation of different durations (***C***). Different shades of grey or blue indicate different stimulation durations. ***D***, Z-scored neuronal responses of all neurons. ***E***, Performance of rats stimulated with light that cannot be sensed by ChR2 (off-wavelength control). ***F***, Performance of rats when stimulating S1 with 40 Hz of 2-ms pulsed blue laser. Download Figure 2-1, TIF file.

10.1523/ENEURO.0453-20.2021.f3-1 Extended Data Figure 3-1 ***A***, Effect of laser stimulation on RT for trials with different stimulation durations. ***B***, ***C***, Cumulative probability plot of the RT of trials with tactile stimuli of different durations (***B***) and trials with both tactile stimuli and laser stimulation of different durations (***C***). Different shades of grey or yellow indicate different stimulation durations. ***D***, Analysis of the “RT” (time of lever release) relative to control vibration (black bar), to laser off (orange bar), and to “laser on” (green bar) for different stimulation durations. Download Figure 3-1, TIF file.

10.1523/ENEURO.0453-20.2021.f5-1 Extended Data Figure 5-1 Laser activation of S1 or corticostriatal neurons could affect neither the movement velocity of the right paw (***A***), nor the overall locomotion speed and distance (***B***, ***C***). ***A***, Velocity of the right paw before and during laser stimulation. Lines represent data for individual session, red line represents mean velocity of all sessions. Bars display mean ± SEM. ***B***, ***C***, Speed of locomotion (***B***) and distance traveled (***C***) before and during laser stimulation. Bars display mean ± SEM. Download Figure 5-1, TIF file.

Simultaneous neural activity was recorded at 25 kHz using a digital headstage (ZD32, TDT). Spike sorting was performed in the KiloSort algorithm (Pachitariu et al., 2016).

### Video recording and tracking

Videos were recorded at 30 fps by seven cameras (6 × acA1300-200uc and 1 × acA800-510uc, BaslerAG) using objectives with focal length 6–8 mm (Kowa), which allowed us to track the rats from different perspectives. To synchronize the laser stimulation with video frames, the acquisition system received TTL pulses when the laser was on and triggered the cameras with another TTL signal (30-Hz square wave with 40-μs width). To track the 3D motion of body parts of rats, a deep learning-based framework, FreiPose, was trained with a number of manually annotated video frames (Zimmermann et al., 2020). Twelve body parts were detected which comprise five points on the head (the snout, the left and right ears and eyes), four limbs, two hind ankles, and the base of the tail.

### Statistical analysis

Statistical analysis between two groups was made using two-sample *t* test. Multiple group comparisons were performed using a repeated-measures ANOVA, which was followed by a *post hoc* Scheffe’s test when significant main effects or interactions were found. Results are expressed as mean ± SEM. Results were considered significant by a probability of *p* ≤ 0.05. Asterisks on figures indicate statistically significant differences. Statistical results are summarized and displayed in Table 1. All statistical analysis was performed with MATLAB programs (The MathWorks Inc.).

**Table 1:** Statistical table

Figure	Trial number	Type of test	Statistics
2	1430	Repeated-measures ANOVA	VT: duration: *p* = 0.0033646 Laser: *p* = 0.0049681 CT: Duration: *p* = 0.06788 Laser: *p* = 0.60458
		Two-sample *t* test	RT: no laser vs laser, *p* = 0.0406
3	3914	Repeated-measures ANOVA	VT: duration: *p* = 0.00013688 Laser: *p* = 0.00058707 50 ms: *p* = 0.017558 CT: duration: *p* = 0.67953 Laser: *p* = 0.72445
		Two-sample *t* test	RT: VT vs VT+laser, *p* = 0.0484
4*A–C*	837	Repeated-measures ANOVA	VT: duration: *p* = 0.0014106 Laser: *p* = 0.00065433 CT: duration: *p* = 0.59919 Laser: *p* = 0.3263
		Two-sample *t* test	RT: VT vs VT+laser, *p* = 0.7963
4*D–F*	5397	Repeated-measures ANOVA	VT: duration: *p* = 1.154 × 10^−6^ Laser: *p* = 0.44539 CT: duration: *p* = 0.0.001413 Laser: *p* = 0.34203
		Two-sample *t* test	RT: VT vs VT+laser, *p* = 0.5430
5*C,D*	2790	Repeated-measures ANOVA	VT: duration: *p* = 0.0061778 Laser: *p* = 0.26705 CT: duration: *p* = 7.755 × 10^−5^ Laser: *p* = 0.012727 75 ms: *p* = 0.0052624 150 ms: *p* = 0.019976 LO: duration: *p* = 0.10267 Laser: *p* = 0.041758 75 ms: *p* = 0.042444 150 ms: *p* = 0.039128
		Two-sample *t* test	RT: VT vs VT+laser, *p* = 0.1126 VT vs LO, *p* = 0.1209 VT+laser vs LO, *p* = 0.0054
5*E,F*	1096	Repeated-measures ANOVA	VT: duration: *p* = 0.35861 Laser: *p* = 0.32564 CT: duration: *p* = 0.86755 Laser: *p* = 0.52315 LO: duration: *p* = 0.0047137 Laser: *p* = 2.8566 × 10^–9^ 75 ms: *p* = 0.00045151 150 ms: *p* = 0.019915
		Two-sample *t* test	RT: VT vs VT+laser, *p* = 0.8524 VT vs LO, *p* = 3.8035 × 10–4 VT+laser vs LO, *p* = 0.0039

## Results

### Behavioral paradigm

To assess the vibration-detecting ability of rats, we designed a vibration detection protocol (Fig. 1). Eleven water-deprived rats were trained to perform the task with their right paw. The rats had to move the lever to the left side and hold it there for 600, 1600, or 2600 ms. After a successful holding time a vibrotactile stimulus was delivered directly to the lever. Rats were rewarded for releasing the lever within an allowed response window of 500–600 ms after this vibration (VTs). We applied four different stimulation durations: 50, 75, 100, and 150 ms. On CTs, the control vibrator attached to a control lever placed outside of the behavioral arena was turned on instead of the real vibrator. To receive a reward in these trials, rats had to hold the lever after the holding period for another 500–600 ms. Afterwards, the reward was delivered. Late responses to VTs (Miss) and incorrect responses in CTs (false alarm) were punished with a 1- to 2-s timeout. The rats performed the task with an average detection rate above 65% (66.4 ± 3.8% in VTs) and a false alarm rate below 40% (37.1 ± 2.5% in CTs).

### Optical activation of S1 partially substitutes vibrotacile stimuli

First, we asked whether optical activation of S1FL can create artificial sensations by substituting real vibrotactile stimuli and in turn induce reactions of the animals. To address this question, we unilaterally injected four Sprague Dawley rats with a viral vector (AAV5-hSyn-ChR2-eYFP) carrying the excitatory opsin ChR2 and implanted optical fibers in left S1FL (Fig. 2*A*). After four weeks, we tested whether blue light stimulation in the injected areas could mimic the tactile vibration stimulus in the absence of vibration and could affect the performance in the presence of vibration (Fig. 2*B*). We applied continuous blue laser light (power 5–10 mW out of a 200-μm fiber, wavelength of 473 nm) of the same duration (50, 75, 100, and 150 ms) as the tactile stimulus. We chose a continuous illumination instead of pulse stimulation to avoid the write-in of a specific frequency. Animals reported the percept in laser-stimulated trials with an average rate of 69.3% [±2.3% (VT+laser); Fig. 2*C*], which is lower than that of unstimulated vibration trials [77 ± 3.2% (VT); repeated-measures ANOVA, factor “duration” *p* = 0.0034, factor “laser” *p* = 0.005]. On the other hand, laser stimulations without vibrotactile stimulation did not lead to a significant increase of responses (repeated-measures ANOVA, *p* = 0.6). These results indicated that laser stimulation which presumably disturbed neural representation of sensory information compromised the perception.

Considering that laser stimulation of different durations yielded similar effects on the behavioral performance, reaction times (RTs) at all durations were pooled together to estimate the effect of laser stimulation on behavior. The analysis of RTs, revealed that laser stimulations significantly reduced the RTs [315.5 ± 9 ms (VT+laser) vs 341.4 ± 8.8 ms (VT), two-sample *t* test, *p* = 0.041; Fig. 2*E*; Extended Data Fig. 2-1*A–C*]. The cumulative distribution frequency plot of RT (Fig. 2*E*) indicated that the faster RTs induced by optogenetic stimulation were because of the percentage increase of short-RT trials (percentage of trials whose RTs were shorter than the median RT of unstimulated trials was 63%, higher than the expected value of 50%). This result is in line with the fact that information written by laser stimulation into sensory cortex is faster than peripheral tactile stimulation. Our electrophysiological recordings revealed that the population neuronal response latency to tactile stimuli was ∼25 ms (Extended Data Fig. 2-1*D*), which is in the same range of change of RT. Further analysis did not reveal any obvious effects of vibration duration or laser duration on the cumulative distribution of RT (Extended Data Fig. 2-1*A–C*). Moreover, the RT of stimulated VT trials with longer holding durations (long holding VT+laser, 291.4 ± 10 ms) was smaller than that of stimulated trials with shorter holding durations (short holding VT+laser, 364.4 ± 15.1 ms, two-sample *t* test, *p* = 6.22 × 10^−5^), and the effect of the laser on RT was stronger for long holding trials (long holding VT, 328.8 ± 10.9 ms, two-sample *t* test, *p* = 0.012; Fig. 2*F*).

A recent study showed that the thermal effect induced by laser stimulation could affect the behavior of mice in the absence of optogenetic actuators (Owen et al., 2019). To exclude the possibility that our results were because of this potential heating effect, we stimulated animals with light that cannot be sensed by the applied opsins (off wavelength control) in a set of control experiments. Yellow light could not alter the behavior of rats expressing ChR2 (Extended Data Fig. 2-1*E*).

Additionally, since it has been shown that continuous illumination leads to desensitization of ChR2 (Nagel et al., 2003), we tested whether pulsed laser light could give rise to stronger effects on behavior. By replacing the continuous laser stimulation with 40-Hz pulses of 2 ms, we observed similar but slightly stronger changes of false alarm rate compared with constant light [1.1 ± 3.2% (continuous) vs 15.8 ± 9.6% (40 Hz), two-sample *t* test, *p* = 0.15; Extended Data Fig. 2-1*F*]. Similarly, long pulsed stimulation compromised the percept and resulted in lower detection rates.

### Optical inhibition of S1 impaired vibrotactile detection

Four Sprague Dawley rats were injected with AAV5-hSyn-NpHR-mCherry into S1FL (Fig. 3*A*). Rats were implanted with optical fibers in the left hemisphere (Fig. 3*A*) and laser light (wavelength of 594 nm, and power of ∼15 mW) was delivered in 50% of trials (Fig. 3*B*). Similar to our findings in the optical activation experiment, the vibration duration significantly affected the performance [44.6 ± 3.1% (50 ms), 72.1 ± 2.5% (75 ms), 81.1 ± 1.7% (100 ms), 87.8 ± 1.9% (150 ms), repeated-measures ANOVA, factor duration, *p* = 0.00014; Fig. 3*C*]. The detection rate increased with longer vibration durations indicating that the rats integrated the tactile input over time with the best performance at 150 ms. Optogenetic inhibition of S1FL disturbed the performance significantly [71 ± 2.5% (VT) to 66 ± 2.1% (VT+laser), repeated-measures ANOVA, factor laser, *p* = 0.00059; Fig. 3*C*]. In contrast, the optical inhibition slightly increased the false alarm rate, although not significantly [from 26.8 ± 1.8% (CT) to 32.7 ± 2.3% (CT+laser), repeated-measures ANOVA, factor laser: *p* = 0.72; Fig. 3*D*], which could have been because of the rebound activity induced by continuous optical inhibition (Kravitz and Bonci, 2013; Häusser, 2014; Li et al., 2019), because the time of lever release relative to “laser off” for a duration of 159 ms was significantly smaller than that for a duration of 50 ms (two-sample *t* test, *p* = 0.02; Extended Data Fig. 3-1*D*), which indicates a stronger rebound activity induced by longer optical inhibition. Additionally, rats displayed significantly longer RTs when S1 was inhibited [increased from 373.8 ± 5.8 ms (VT) to 389.6 ± 5.4 ms (VT+laser), two-sample *t* test, *p* = 0.0484; Fig. 3*E*]. Furthermore, the RT change induced by optical inhibition can be explained by the percentage decrease of short-RT trials (percentage of trials whose RTs were shorter than the median RT of unstimulated trials was 43%, smaller than the expected value of 50%). Thus, the transient optical inhibition of S1 impaired the vibration detection, consequently, it took longer for the rats to accumulate sensory evidence.

We further investigated how the stimulus duration and holding duration affected the RT. RT distributions were affected neither by the vibration duration (ANOVA, factor duration: *p* = 0.12, factor laser: *p* = 0.02; Extended Data Fig. 3-1*A–C*) nor by holding duration (ANOVA, factor “holding duration”: *p* = 0.61, factor laser: *p* = 0.03; Fig. 3*F*).

### Striatal terminal manipulation and cortical manipulation differentially affected behavior

To probe the role of cortical somatosensory projections to striatum in our behavioral protocol, we optically activated or inhibited the axons of neurons in S1FL projecting to striatum (Fig. 4*A*,*D*). We manipulated the terminals in dorsolateral striatum (DLS) which receives direct input from S1FL (Extended Data Fig. 1-1*B*; McGeorge and Faull, 1989; Brown et al., 1998; Hoover et al., 2003; Hooks et al., 2018; Hidalgo-Balbuena et al., 2019). The combination of tactile and optogenetic stimuli resulted in a significantly increased detection rate [increase from 77.9 ± 4.1% (VT) to 83.9 ± 1.8% (VT+laser), repeated-measures ANOVA, factor duration: *p* = 0.0014, factor laser: *p* = 0.0007; Fig. 4*B*, left panel]. The response rate to striatal optogenetic stimulation (CT+laser) tended to slightly increase compared with CTs, although not significant (repeated-measures ANOVA, factor laser: *p* = 0.33; Fig. 4*B*, right panel). To assess the effect of terminal inhibition on behavior, we implanted optical fibers in the ipsilateral striatum (Fig. 4*C*). In contrast to optical inhibition of S1FL, neither the detection rate nor the false alarm rate was affected by optical inhibition of terminals in striatum (Fig. 4*D*).

### Striatal terminal manipulation and corticostriatal neuron manipulation differentially affected behavior

Since sensory cortex projects to various brain regions and some of these projections might pass through striatum, we asked whether laser stimulation of striatum-projecting neurons in S1FL and their terminals in striatum yielded different effect on behavior compared with unspecific stimulations of S1FL and projections in striatum, respectively. Moreover, considering that neurons projecting to striatum might send collaterals to other structures and that there could be antidromic activation of S1FL neurons when stimulating terminals, we next asked whether manipulating neurons in S1FL projecting to striatum might cause differential effects compared with the manipulation of the striatal terminals and the general manipulation of S1FL neurons. For this, we targeted the corticostriatal pathway by injecting a Cre-dependent AAV5-ChR2-eYFP in S1FL and a Cre-carrying retrograde AAV (rAAV2-retro-Cre) in ipsilateral DLS (Fig. 5*A*). We additionally stimulated in 50% of the trials S1FL with blue laser light, in which responses to the laser were rewarded (Fig. 5B). Half of these stimulated trials contained both vibration and laser stimulation (VT+laser; Fig. 5*B*). We further provided blue laser light in 50% of CTs, in which responses to the laser were not rewarded (CT+laser). Activation of corticostriatal neurons slightly decreased the detection rate of tactile stimuli [decreased from 66.7 ± 1.99% (VT) to 58.9 ± 2.1% (VT+laser); repeated-measures ANOVA, factor duration, *p* = 0.0062; factor laser, *p* = 0.27; Fig. 5*C*, left panel], and laser stimulation evoked reactions in CTs [increased from 31.4 ± 1.7% (CT) to 56.4 ± 3.8% (CT+laser); repeated-measures ANOVA, factor laser, *p* = 0.013; Fig. 5*C*, right panel]. The RT was not affected significantly by the optical activation of corticostriatal cell bodies when the vibration was presented [404.6 ± 7.6 ms (VT), 425.6 ± 10.4 ms (VT+laser), two-sample *t* test, *p* = 0.11; Fig. 5*D*]. Surprisingly, optical activation of terminals did not alter the performance significantly (Fig. 5*E*, left panel). Further, terminal activation had no effect on RT when the vibration was presented (Fig. 5*F*).

Because of the strong effect of laser stimulation in CTs, we aimed to evaluate the effect of pure laser stimulation on performance. For this, some trials were stimulated with laser only (LO) without vibration or control vibration stimuli (Fig. 5*B*). We found that optical activation of corticostriatal cell bodies could evoke responses on trials with laser stimulation only [31.4 ± 1.7% (CT) vs 55.6 ± 2.3% (LO), repeated-measures ANOVA, factor duration, *p* = 0.1; factor laser, *p* = 0.041; Fig. 5*C*, right panel]. Furthermore, animals responded faster on trials where only laser stimulation [383.3 ± 10.9 ms (LO)] was presented than those with both laser and vibration [425.6 ± 10.4 ms (VT+laser), two-sample *t* test, *p* = 0.0054; Fig. 5*D*]. This result indicated that information written in by laser stimulation was differently perceived than that written in by vibrotactile stimulation, regardless of the presence of laser. We speculate that the vibrotactile stimulus overwhelmed the effect induced by laser stimulation. Therefore, laser stimulation in the absence of vibrotatile stimulus could lead to faster reactions. Intriguingly, although optical activation of corticostriatal terminals did not significantly affect the detection of vibration (Fig. 5*E*, left panel), optical activation evoked significantly more responses (repeated-measures ANOVA, *p* = 2.9 × 10^−9^), and short optical activation of corticostriatal terminals was more efficient in evoking responses [52.8 ± 2.5% (LO, 75 ms), *post hoc* test, *p* = 0.00045] than long optical stimulation [39.7 ± 2.7% (LO, 150 ms), *post hoc* test, *p* = 0.02; Fig. 5*E*, right panel] on LO trials. Similarly, optical activation of corticostriatal terminals led to faster RTs in the absence of vibration [395.3 ± 8.4 ms (VT), 392.4 ± 13.4 ms (VT+laser), 331.9 ± 15.8 ms (LO); VT vs LO: two-sample *t* test, *p* = 3.8 × 10^−4^; VT+laser vs LO: two-sample *t* test, *p* = 0.0039; Fig. 5*F*].

### Optical stimulation in the open field

As optogenetic stimulation of barrel cortex triggers whisker movements (Matyas et al., 2010; Auffret et al., 2018), we asked whether optogenetic stimulation of paw-related somatosensory cortex provokes forelimb movements. To answer this question is important to ensure that optical stimulations can indeed elicit a biomimetically relevant sensation and that the responses observed in our experiment were not caused by paw movements induced by the stimulation of sensory cortex. To address this point, we stimulated S1FL when the rats were moving freely in an open-field arena. Each rat was video-recorded by seven video cameras from different perspectives, and the movements of different body parts were extracted off-line using a machine-learning based video tracking algorithms (Zimmermann et al., 2020). To get a comprehensive assessment of the behavior, we performed kinematic analysis of the right paw. We did not observe any change in paw velocity caused by laser stimulation, indicating that the laser stimulation did not trigger paw movements (Extended Data Fig. 5-1*A*). These results support the claim that the optogenetic stimulation was perceived as sensory perception and only in turn initiated a response.

A recent study showed that activation of corticospinal neurons in S1 could regulate locomotion (Karadimas et al., 2020). To investigate whether the manipulation of corticostriatal neurons in S1 could influence spontaneous behavior, we performed a similar experiment. We found that the manipulation of these neurons did not change the locomotion (Extended Data Fig. 5-1*B*,*C*), except that stimulation consisting of 5-ms pulses at 30 Hz for 5 s with an interstimulus interval of 15 s caused a freezing of the right paw as well as a backward stepping movement. As we did not use this stimulation frequency for our tactile perception experiment it seems unlikely that this effect affected our study.

In summary, our current study provided insights into neural circuits involved in vibrotactile perception, and revealed the importance of S1 and the corticostriatal pathway in the performance of tactile detection task. Furthermore, our results indicate that S1 and its projections to striatum encode different domains of tactile perception. Moreover, our open field control experiment suggests that the optogenetic stimulation caused a perception as opposed to a direct motor output.

## Discussion

By bidirectionally modulating neural activity of neurons in S1 and its projections to striatum in freely-moving rats during a vibration detection task using optogenetic tools, we found that S1 convey essential signals that drive the performance in vibration detection. However, S1 seems to be a less suitable candidate to write in artificial information as the detection of tactile inputs gets disturbed by the laser manipulation. In contrast, activation of sensory terminals in striatum improved the performance in the presence of vibration. In an effort to investigate the relative contribution of this particular neural pathway, we used a dual-virus system and found that activation of the corticostriatal pathway gave rise to the strongest effects on performance, especially in the absence of tactile stimuli. Thereby, our study revealed that the S1-striatum pathway causally participates in sensory detection and that the corticostriatal projection neurons might be a preferable target for a sensory write in.

Our results implied the participation of the sensory network in the execution of the task and suggest that optogenetic stimulation of neurons in corticostriatal pathway could partially substitute tactile stimuli without eliciting paw movements. In contrast, a previous study reported that neurons in somatosensory cortex preferentially targeting D1 direct pathway neurons trigger actions (Wall et al., 2013). However, neurons in whisker-related S1 innervate parvalbumin (PV)-expressing interneurons as well (Lee et al., 2019); therefore, S1FL might have the potential to improve performance by suppressing undesired actions. Additionally, this study found that optogenetic stimulation of S1 corticostriatal afferents led to less responses in the tactile discrimination task, which is different from our results. These disparities could be because of the physiological difference between whisker and forelimb system, which is discussed below.

In optogenetic experiments, it is important to ensure that animals do not use the laser light itself as inadvertent cue to perform the task. There are several ways to exclude this possibility, such as laser stimulation without opsin expression (eYFP/mCherry control in our case), control experiments with the optical fibers disconnected, and stimulations with light that does not activate the opsins (off-wavelength control; May et al., 2014; Kressel et al., 2020). Laser stimulation without opsin expression is the most commonly used control, since all conditions are the same except that there is no expression of opsins. In our study, we used the stimulation with another wavelength that cannot be sensed by opsins as a control and found no behavioral change, which indicated that animals did not purely use the laser light as an additional cue. This control has its limitations. The wavelength of the laser was different in optogenetic and control experiments which might have led to differences in thermal effects with higher temperature increases induced by blue light (Stujenske et al., 2015; Arias-Gil et al., 2016) as well as in differences in visual detectability of the laser light with preferences for the blue spectrum (Walton, 1933). However, both the thermal as well as the detectability factor are in favor of introducing a behavioral effect independent of opsins using blue light, therefore providing a rather conservative control with the advantage of intraindividual comparisons.

In our study, optical inhibition of terminals in striatum did not result in any behavioral change. This might be partially explained by technical issues, i.e., the complexity in silencing synaptic terminals (Mahn et al., 2016; Wiegert et al., 2017) or inadequate coverage of viral infection or light. Most likely we did not shut down the complete information pathway between S1FL and striatum. Alternatively, sensory information carried in other pathways rather than the corticostriatal pathway could have exerted the sensory detection during terminal inhibition. For instance, it is possible that striatum-projecting neurons in S1 send collaterals to other brain areas that participate in this task, such as primary and secondary motor cortex (Zakiewicz et al., 2014). In this scenario, when the S1-striatum pathway is inhibited, other neural pathway would kick in to initiate the movement. Moreover, the lack of behavioral effects on inhibition of corticostriatal terminals could putatively be explained by information redundancy. It has been suggested that information redundancy exist ubiquitously in neural system (Chechik et al., 2006). Furthermore, it has been demonstrated that the number of neurons carrying information relevant to the task is much higher than that of neurons accounting for the performance of the task (Britten et al., 1992).

Anatomically, many striatum-projecting brain regions, such as the posteromedial complex (POm) and parafascicular thalamic nucleus (Pf) of the thalamus (Alloway et al., 2017), send projections to S1 as well (Ohno et al., 2012; Alloway et al., 2017; Unzai et al., 2017; Zhang and Bruno, 2019). Previous studies have shown that neural responses of DLS neurons occurred either before or at the same time as those of neurons in S1 and that the speed of adaptation in S1 is faster than in DLS. Further, S1 neurons only respond to the initial phase of back-and-forth whisker deflections while DLS neurons exhibited two response to both forward and backward deflections of the whisker stimulation (Ahissar et al., 2001; Khatri et al., 2004; Melzer et al., 2006; Chakrabarti and Alloway, 2009; Alloway et al., 2017), indicating that sensory information represented in DLS must, at least partially, arise from additional subcortical regions (Mowery et al., 2011; Smith et al., 2012; Alloway et al., 2014). In terms of the function of this subcortical pathway, it has been suggested that subcortical transmission to striatum could convey modality-specific information required for selection and initiation of a particular sensory-guided response (Smith et al., 2012). However, it remains elusive how the sensory representation in DLS contributes to the performance of sensory discrimination task and whether various projections exert diverse impacts on information encoding in DLS. In our hands, activation of corticostriatal neurons disturbed the performance, while corticostriatal terminal activation not only evoked a perception, but also slightly improved the performance, which indicates that this pathway plays a causal role in the detection task.

Additionally, when this corticostriatal pathway is activated, the antidromic activity could activate S1 neurons, which in turn could activate task-relevant brain regions receiving input from S1. Extensive studies identified reciprocal connections between POm and S1 (Nothias et al., 1988; Hoffer and Alloway, 2001; Aronoff et al., 2010; Smith et al., 2012). Further, S1 sends collaterals to POm (Smith et al., 2012) and striatum (Lévesque et al., 1996; Kita and Kita, 2012; Watakabe et al., 2014).Thus, same S1 neuron may send collaterals to different downstream brain regions. Nevertheless, in our pathway-specific activation experiment, activation of corticostriatal neurons versus activation of striatal terminals yielded different results, which indicates that the antidromic activation of S1 neurons plays a minor role. Our finding that activation of corticostriatal neurons disrupted performance implies that these S1 neurons might be also involved in the sensory presentation. Taken together, these results indicate that direct and indirect projections from S1 to striatum via the corticostriatal and subcortical pathway may have different roles in the vibration detection task.

It has been demonstrated that basic motor patterns of the paw were not perturbed by S1FL photoinhibition (Mathis et al., 2017). In the current study our open-field experiment demonstrated that S1 activation with the chosen parameters had no effect on paw movement either. These results are puzzling, especially considering the existence of corticospinal neurons in S1 (Coulter and Jones, 1977; Karadimas et al., 2020). In contrast to our findings, it has been revealed that photoactivation of barrel cortex could trigger whisker movement (Matyas et al., 2010; Sreenivasan et al., 2015; Auffret et al., 2018), while photograph inhibition of barrel cortex reduces the vigor of whisker movements (Hong et al., 2018) and perturbation of barrel cortex activity could affect whisking (Matyas et al., 2010). This inconsistency of behavioral effects observed between S1FL and barrel cortex might be because of the fact that whisker movements can be detected more easily, whereas the delicate paw movement change is hard to notice or minor muscle activity change will not result in movement change at all. Additionally, the differences could also be attributed to the anatomic difference between the whisker system and the paw system. Unlike muscles involved in paw movement, intrinsic muscles driving whisker movements do not have spindles for proprioception. Therefore, the motor control by barrel cortex might provide important sensory input to the whisker motor system (Sreenivasan et al., 2015). In conclusion, the inefficiency of stimulation of S1FL in evoking paw movements indicates that the direct role of sensory cortex in motor control may be a specialization of the whisker system, rather than a general feature of sensory cortex. Taken together, our current work provides important insights into how different neural networks involving S1 contribute to vibration-triggered movement initiation, which will facilitate a more comprehensive view of the functions of sensorimotor system, and highlighted the potential of the corticostriatal circuit in sensory restoration.

## Synthesis

Reviewing Editor: Alexxai Kravitz, Washington University in St. Louis Decisions are customarily a result of the Reviewing Editor and the peer reviewers coming together and discussing their recommendations until a consensus is reached. When revisions are invited, a fact-based synthesis statement explaining their decision and outlining what is needed to prepare a revision will be listed below. The following reviewer(s) agreed to reveal their identity: Sebastiano Bariselli, Tanya Sippy.

1) The comment on statistical analyses made by Reviewer 1 should be addressed by RM ANOVA with proper post-hoc tests as suggested by Reviewer 1. This is a critical comment as it relates to the robustness of the main findings.

2) Multiple comments from both reviewers related to clarity of the description of the experimental design should be addressed fully.

3) For striatal terminal activation/inactivation was same power used? Given challenges of optogenetic terminal inhibition (Mahn M., Prigge M., Ron S., Levy R. & Yizhar O. (2016) Nature Neuroscience. 19, 4, p. 554-6), how are the authors certain they successfully inhibited terminals when no effects observed behaviorally?

4) Additional discussion should address why they think their effects only occurred at certain stimulation lengths.

5) Histology is very minimal and limited to one image of viral expression in Figure 1-1. This image could be moved to a main figure, and if possible larger attempts should be made to demonstrate proper expression in all behavioral conditions.

In addition, the full reviewer comments are below. Reviewer #2 does ask for additional GFP controls that would likely require new experimentation. This is not critical for a resubmission, although the limitation brought up by the reviewer should be discussed.

****************************

Reviewer #1

****************************

In this manuscript, the authors report the effects of sensory pathway (S1FL-striatum) stimulation and inhibition on the performance of a vibrotactile perception-based behavioral task in rats. The authors are attempting to understand whether stimulation and inhibition of either cell bodies, corticostriatal terminals and projection-specific cell bodies have similar effects on task performance. While the author’s efforts in exploring this outstanding question, that goes beyond the sole understanding of the effects of optogenetics on corticostriatal circuits, are much appreciated, their data are strongly undermined by inappropriate and unclear statistical analysis. The authors should first clarify why they perform a two-way ANOVA instead of a RM two-way ANOVA. The latter seems to be the most appropriate as multiple laser stimulations of different durations are delivered within the same subjects. Moreover, after ANOVA, they often apply unpaired two-tailed t-test that does not correct for multiple comparisons (e.g. Figure 2D, Figure 3D). The authors are encouraged to perform appropriate statistical analysis to ensure a clear understanding and interpretation of these results. Although the graphical representation is good and the manuscript is easily readable, I recommend major revisions before publication.

Other comments:

- For replicability purposes, the authors should better clarify whether rats were also food deprived.

- The authors should clarify the rationale for the experiments reported in Figure 5. I believe that with those experiments the authors are investigating whether the stimulation of cell bodies of striatal-projecting S1FL neurons has different outcomes than the stimulation of the entire S1FL population. In fact, even with retrograde virus infusions, they cannot exclude the activation of neurons that send collaterals to other brain regions other than the striatum.

- For replicability reasons, the authors should report the duration of the training needed to reach a 60% success rate, which is then used as a criterion to proceed with the optogenetic experiments.

- The authors should specify whether they included reaction times at all laser duration or only at specific laser duration, and provide a justification.

- In the experiments on S1FL inhibition, the authors reported an improved performance at increasing vibration duration. However, they found a reduced performance at short laser stimulation, compared to longer ones. Would this effect be due to a rebound excitation that overlaps with vibrotactile stimulation?

- In Figure 3D, the authors report an increased responding at long-stimulation duration. If this effect was due to rebound activation of S1, wouldn’t the direction of the change be a decrease as in the optogenetic excitation experiments (Figure 2D)?

- In Figure 5C, the authors should add a legend to clarify what groups they are comparing.

- In Figure 5D, the authors report similar reaction times (RT) for VT and VT+lasers. However, they also report a significant reduction in RT in laser stimulation-only (LO) group compared to VT+laser. The authors are encouraged to comment on that: is this because the laser feeds completely different information into the corticostriatal network, compared to VT or VT+laser? Is the vibrotactile perception changing how rats perceive the simultaneous laser stimulation?

****************************

Reviewer #2

****************************

In this paper, the authors seek to understand what impact optogenetic stimulation has on somatosensory perception. This question has been addressed in monkeys and rodents in several prior studies cited by the authors and they seek to further this work by using cell type specific activation and inactivation, specifically of corticostriatal neurons from S1 to striatum to understand if this subpopulation has a specific, definable role.

Major comments

• please clarify what is the difference between ’control vibrator" and "real vibrator" This only becomes clear in the figure legend of figure 1 that there is no vibration delivered by the "control vibrator" - wording here is confusing - why would you call it a control vibrator when no vibration is being given at all?

• it is unclear in the authors description of the task why 3 discreet times, 0.6, 1.6 and 2.6s after trial onset are used before delivery of the stimulus - I would think a randomly chosen time between some interval would control better for any learning of the the time delay that could contribute to lever release.

• It is also unclear from task description why 3 different lengths of the stimulus are used in the initial decription of the behavior although it becomes more clear when authors describe figure 2. I would it would be helpful to plot psychometrics of this in figure 1 and text.

• important controls are missing - expression of GFP or YFP without opsins for both excitation and inhibition experiments. Authors show that yellow wavelength of light does not yield behavioral effects but given different color not best control for effect of blue light at time of stim presentation

• the authors do not mention in the methods which coordinates they use for retro-AAV injections - striatum is a large structure and has topographic representation of different sensory areas. It is important that authors target correct location. I suggest they cite or demonstrate projections from S1FL to DLS and show they target this region.

Minor comments

• authors miss some key citations - specifically when citing projections from S1 to striatum - see work from Petersen lab (Sippy, et al 2015) and Hinitriyan, et al 2016

• why did the authors train before surgery and then retrain after surgery? would be nice to have some explanation here.

• please clarify length of laser stimulation used in the text in experiments shown in fig 2C

• Typo in figure legend 1 - should read "were not reward(ed)

• I think there is a mistake in figure 2B - legend and text say stim in 20% trials but figure states 50%

• please specify wavelength of light used in text section 3 when decribing optical inhibition for ease of read

• if authors think increased FA rate with rebound activity, could analyze the time of lever release to see how is coincides with laser off.

• not sure I agree with statement: "the opposing effects...indicate animals did not use light as an additional cue." They still could use light in combination with presence of absence of vibration in a combinatorial way that is not as obvious as this interpretation

• is inset in fig 3E across all holding durations or only the longest?

• in panel 3E it seems as if RT is significantly increased with laser but this does not seem to be true for any of the 3 holding durations shown in 3F - please clarify

• for striatal terminal activation/inactivation was same power used? How are the authors certain they successfully inhibited terminals when no effects observed behaviorally?

• how do the authors make sense of the fact that corticostriatal terminal activation increases response rate at some stim lengths (50 and 100) but not others (75 and 150)?
