# Supplementary figures and images for: Effects of Optogenetic Stimulation of Primary Somatosensory Cortex and Its Projections to Striatum on Vibrotactile Perception in Freely Moving Rats
Source: eNeuro. 2021 Mar 5;8(2):ENEURO.0453-20.2021. doi: 10.1523/ENEURO.0453-20.2021 (PMC7986534; doi:10.1523/ENEURO.0453-20.2021)

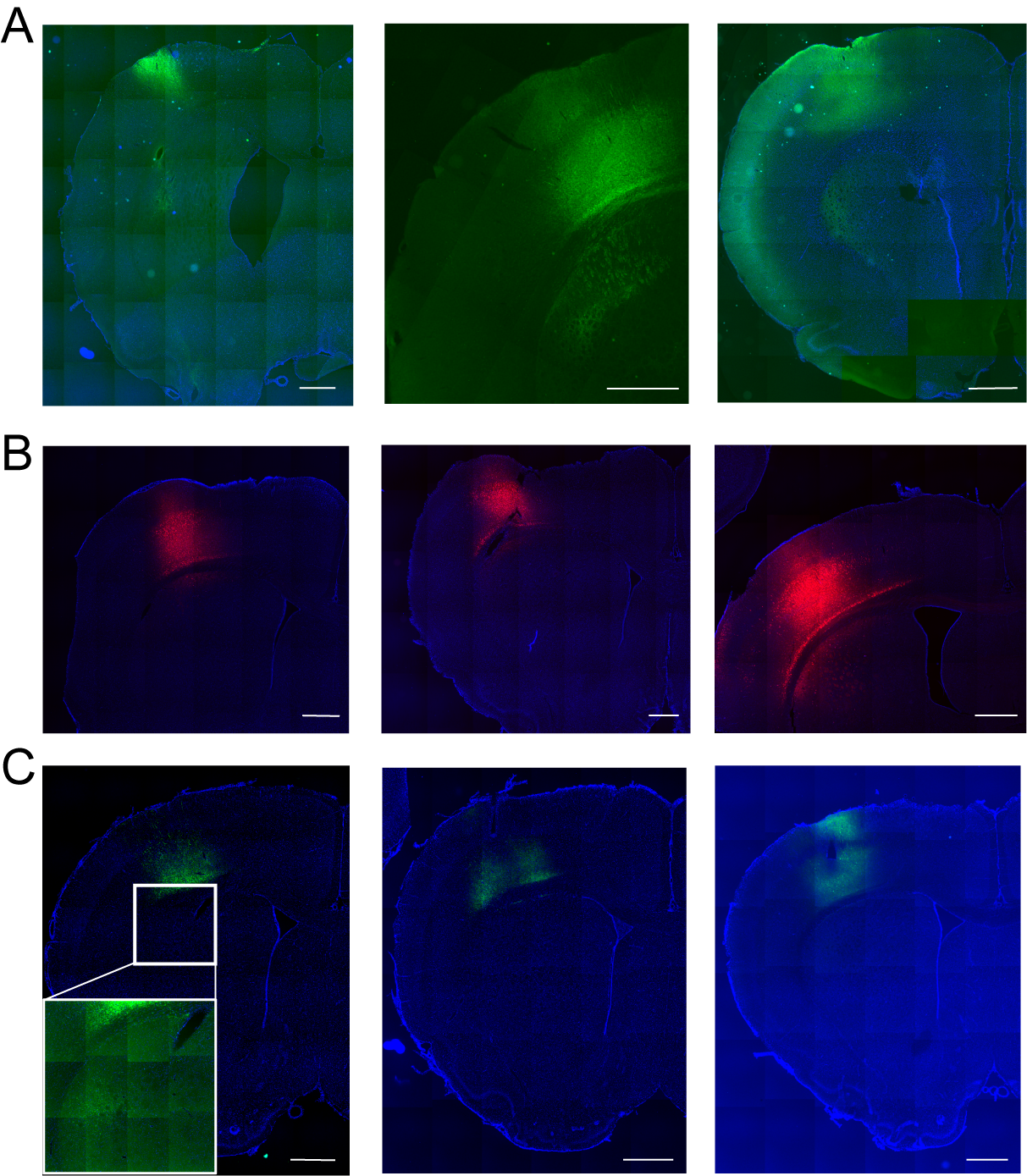

Supplement: Extended Data Figure 1-1 — Histological verification of opsin expression in S1FL of a coronal slice from rats involved in optical activation (A), inactivation (B), and pathway-specific activation (C) experiments, respectively. Scale bar: 1 mm. The inset represents an enlarged, higher magnification of the boxed region. Fibers are visible in striatum. Download Figure 1-1, TIF file. [file enu-eN-NWR-0453-20-s03.tif]

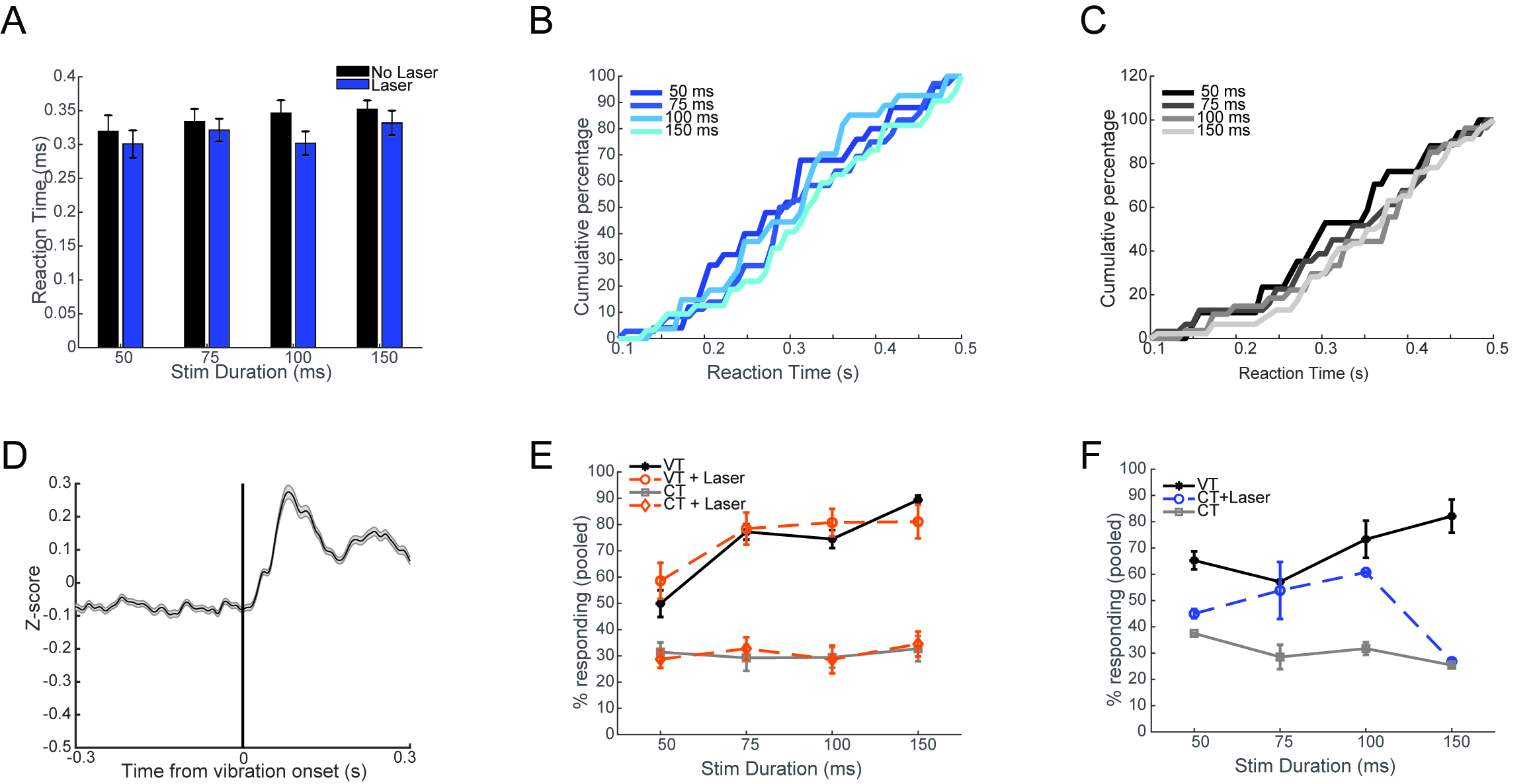

Supplement: Extended Data Figure 2-1 — A–C, Effect of durations of optical activation in S1on RT. A, Effect of laser durations on RT. B, C, Cumulative probability plot of the RT of trials with tactile stimuli of different durations (B) and trials with both tactile stimuli and laser stimulation of different durations (C). Different shades of grey or blue indicate different stimulation durations. D, Z-scored neuronal responses of all neurons. E, Performance of rats stimulated with light that cannot be sensed by ChR2 (off-wavelength control). F, Performance of rats when stimulating S1 with 40 Hz of 2-ms pulsed blue laser. Download Figure 2-1, TIF file. [file enu-eN-NWR-0453-20-s04.tif]

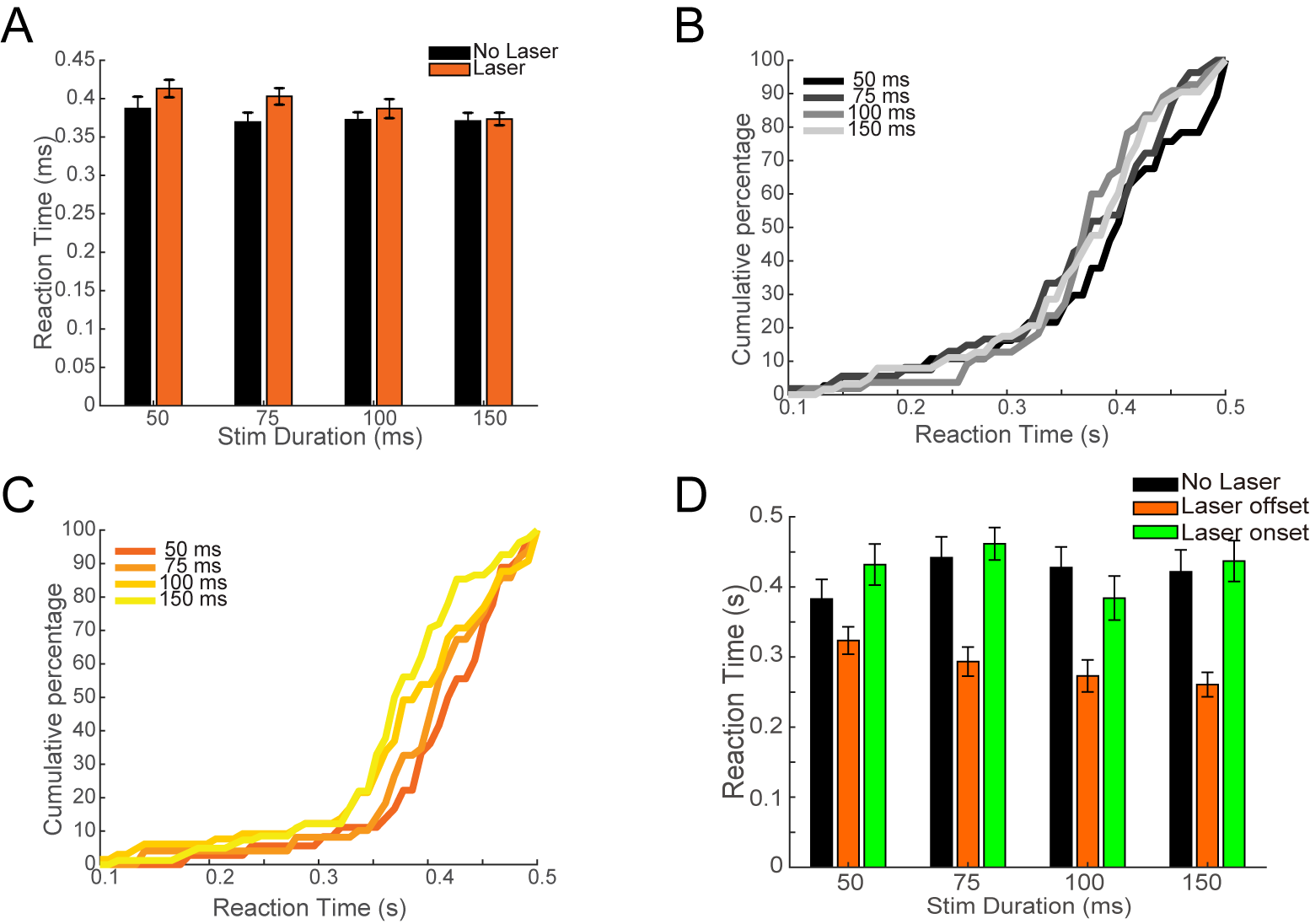

Supplement: Extended Data Figure 3-1 — A, Effect of laser stimulation on RT for trials with different stimulation durations. B, C, Cumulative probability plot of the RT of trials with tactile stimuli of different durations (B) and trials with both tactile stimuli and laser stimulation of different durations (C). Different shades of grey or yellow indicate different stimulation durations. D, Analysis of the “RT” (time of lever release) relative to control vibration (black bar), to laser off (orange bar), and to “laser on” (green bar) for different stimulation durations. Download Figure 3-1, TIF file. [file enu-eN-NWR-0453-20-s01.tif]

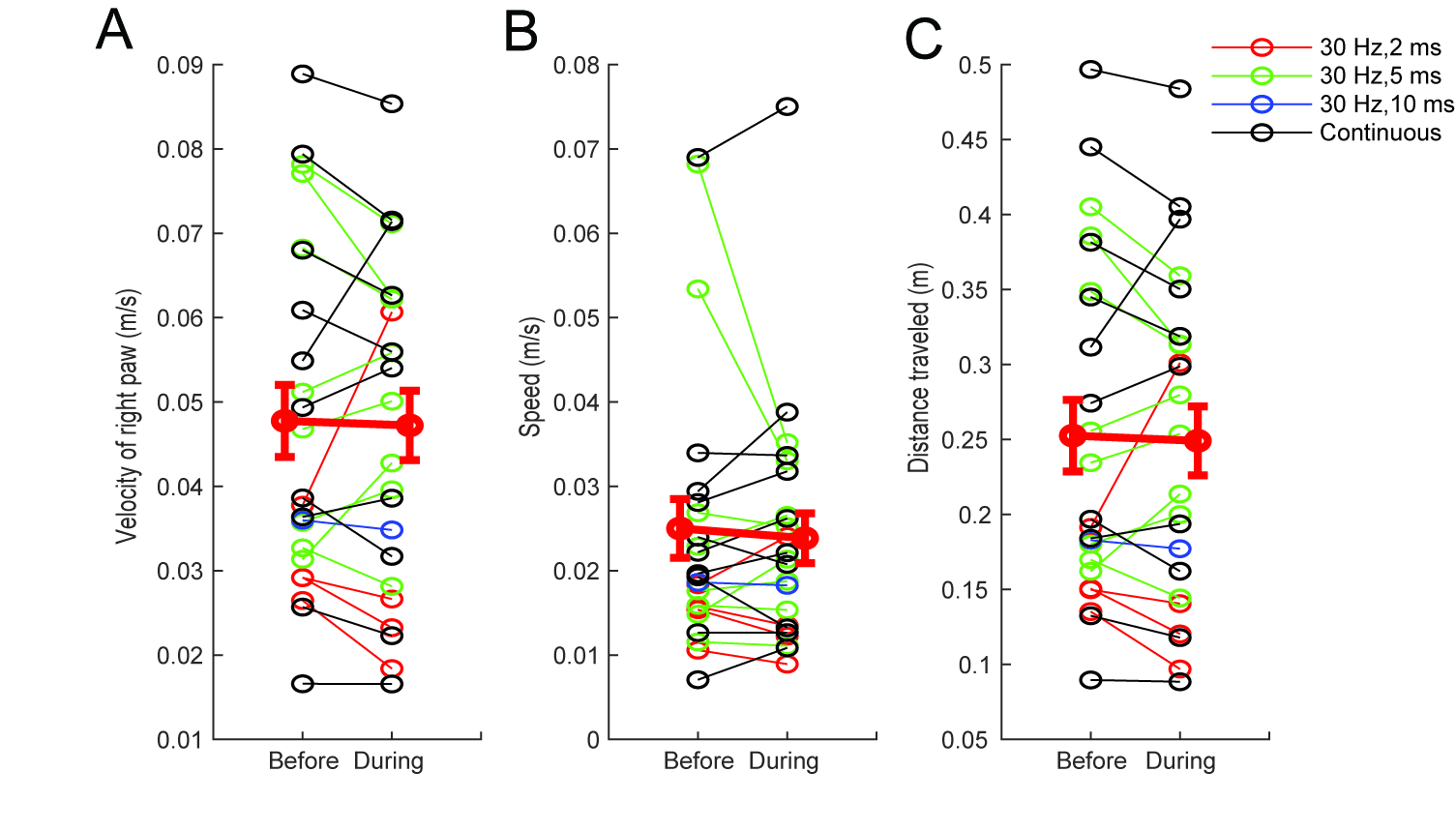

Supplement: Extended Data Figure 5-1 — Laser activation of S1 or corticostriatal neurons could affect neither the movement velocity of the right paw (A), nor the overall locomotion speed and distance (B, C). A, Velocity of the right paw before and during laser stimulation. Lines represent data for individual session, red line represents mean velocity of all sessions. Bars display mean ± SEM. B, C, Speed of locomotion (B) and distance traveled (C) before and during laser stimulation. Bars display mean ± SEM. Download Figure 5-1, TIF file. [file enu-eN-NWR-0453-20-s02.tif]
